# A Review on Artificial Intelligence as a Solution to Burnout in Interventional Radiology

**DOI:** 10.1007/s00270-026-04440-4

**Published:** 2026-04-24

**Authors:** Henry Zhang, Aria Torkpour, Boroumand Zeidaabadi, Nader Ashraf, Anousha Yazdabadi, Shams I. Iqbal, Hamed Asadi, Roberto Luigi Cazzato, Robert Morgan, Behnam Shaygi

**Affiliations:** 1 Imperial College London, London, SW7 2AZ UK; 2 College of Medicine, Alfaisal University, Takhasusi Road, P.O. Box 50927, Riyadh, Saudi Arabia; 3 Eastern Health Clinical School, Monash University and Eastern Health, Melbourne, Australia; 4 Massachusetts General Hospital, Boston Massachusetts, MA 02114 USA; 5 Neurointerventional Radiology Unit, Monash Health, Melbourne, Australia; 6 School of Medicine, Deakin University, Waurn Ponds, Geelong, Australia; 7 Department of Interventional Radiology, University Hospital of Strasbourg, Strasbourg, France; 8 St George’s University Hospitals NHS Foundation Trust and St George’s, Blackshaw Road, London, SW17 0QT UK; 9 London North West University Healthcare NHS Trust, A404 Watford Rd, Harrow, HA1 3UJ UK

**Keywords:** Artificial intelligence, Interventional radiology, Burnout

## Abstract

**Purpose:**

We review burnout risk factors in interventional radiology (IR) and explore how artificial intelligence (AI) would address burnout from a workplace aspect.

**Materials and Methods:**

We performed a literature search on PubMed on risk factors for burnout in interventional radiology and AI tools to address burnout challenges.

**Results:**

IR specialists face burnout risk at personal, workplace and system levels. AI could identify burnout using demographic data and free text, alleviate administrative workload, and manage workflow. AI could also enhance procedural efficiency via automated navigation systems, reducing stress from radiation exposure. Future directions include enhanced burnout identification and medical coding for access to longitudinal data.

**Conclusion:**

AI may be a solution to addressing specific burnout risk factors in interventional radiology.

**Level of Evidence:**

No level of evidence. Review Article.

**Graphical Abstract:**

**Figure:**
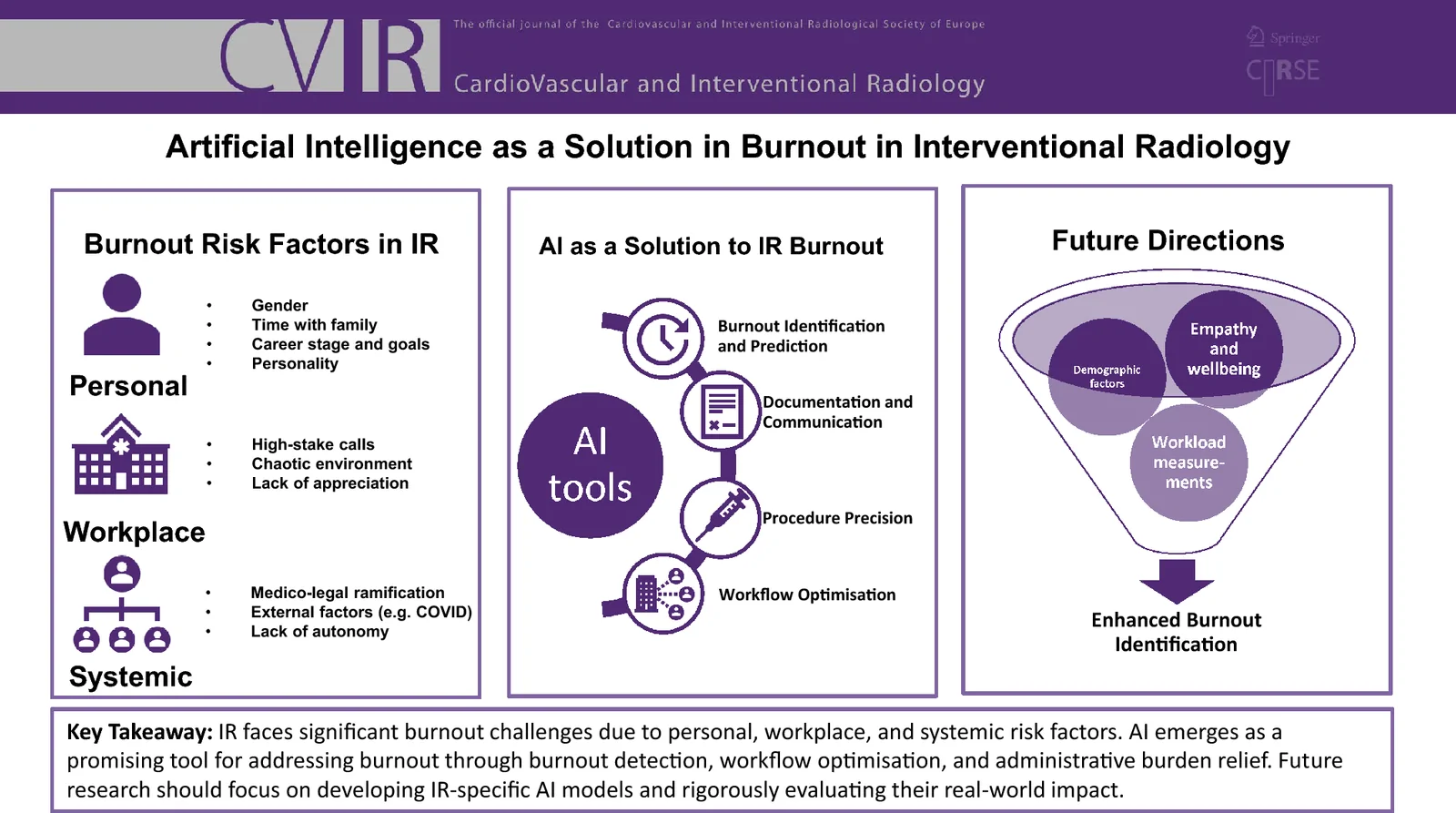

## Introduction

Burnout is a growing concern among interventional radiology (IR) professionals, with an incidence rate of up to 76.7% [1]. Burnout is characterised by emotional exhaustion, depersonalisation, and reduced personal accomplishment, with significant consequences for patient care, healthcare costs, and clinician well-being [2]. It extends beyond a subjective psychological experience, with physiological manifestations including an association with autonomic nervous system function, particularly reflected in heart rate variability (HRV) [3], which contribute to increased cardiovascular risks [4]. The multifaceted nature of burnout underscores the need for comprehensive approaches to its assessment and management, particularly in high-stress specialities like IR.

The Maslach Burnout Inventory (MBI), the gold standard for burnout diagnosis [1, 5], has been widely applied to identify burnout in IR [1, 6]. Consensus is lacking on the dichotomous cutoff of burnout based on the MBI, limiting comparability across burnout research [7, 8]. The conventional MBI-based burnout definition is limited by its overreliance on emotional exhaustion and depersonalisation, with personal accomplishment factors being often overlooked [7–9]. Knowledge gaps in the causes of burnout render methods targeting the three domains of the MBI ineffective [5]. The Copenhagen Burnout Inventory, intended for generic burnout assessment, has shown predictability in overall health, painkiller use, and intention to quit [10]. There is a need to map a clearer symptom aetiology correlation.

**Fig. 1:**
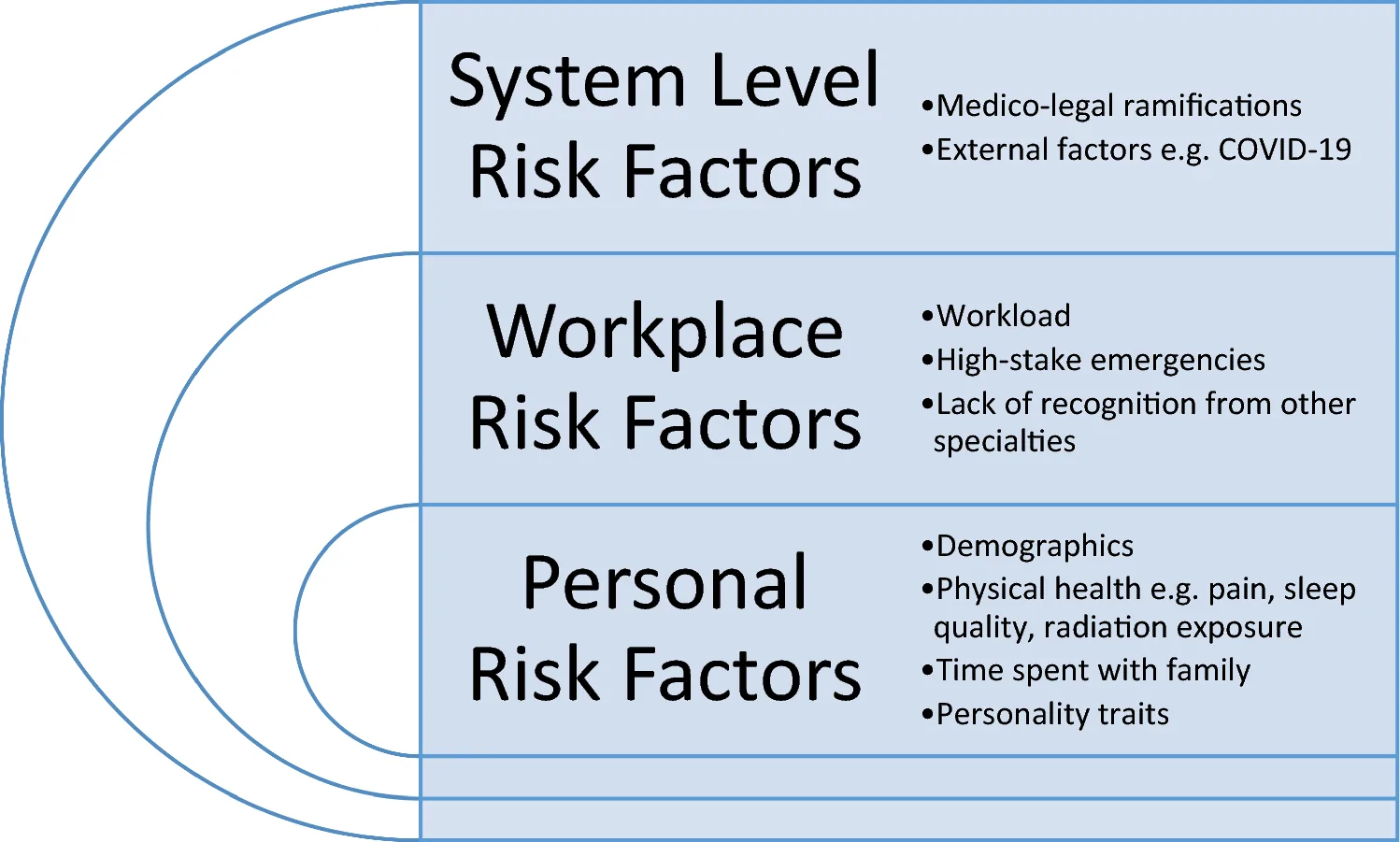
Summary of burnout challenges in interventional radiology at different levels. Burnout risk factors in interventional radiology can be classified in 3 categories: personal, workplace-related, and system level. Personal risk factors include demographics (e.g. female), physical health (e.g. ergonomic pain, sleep quality and radiation exposure), reduced family time, and certain personality traits. Workplace-related risk factors include workload, high-stakes call burden, and lack of recognition from colleagues. System-level risk factors include malpractice, medicolegal ramifications, and external risk factors (e.g. COVID-19)

Artificial intelligence (AI) has emerged as a promising tool for understanding and mitigating burnout in healthcare with three main action pathways:

1. identifying burnout sources at individual (e.g. demographics including sex), workplace (e.g. inter-specialty communication), and systematic levels (e.g. career stage) (Fig. 1) [11, 12];
2. predicting individual burnout risk with demographic data, work-related parameters (e.g. electronic health records (EHRs) and clinical notes), and wearable signals (e.g. HRV) [13, 14],
3. increasing productivity to relieve workload and stress [11].

This literature review aims to explore IR burnout risk factors, and the roles of AI in addressing them. While acknowledging multifactorial sources of burnout among IR specialists including personal health, family life, and financial issues [1, 15, 16], this review intends to explore how burnout could be tackled from a workplace aspect. By examining existing research and identifying future directions, we hope to inform the development of effective strategies for promoting IR well-being. 

### Literature Selection

**Fig. 2:**
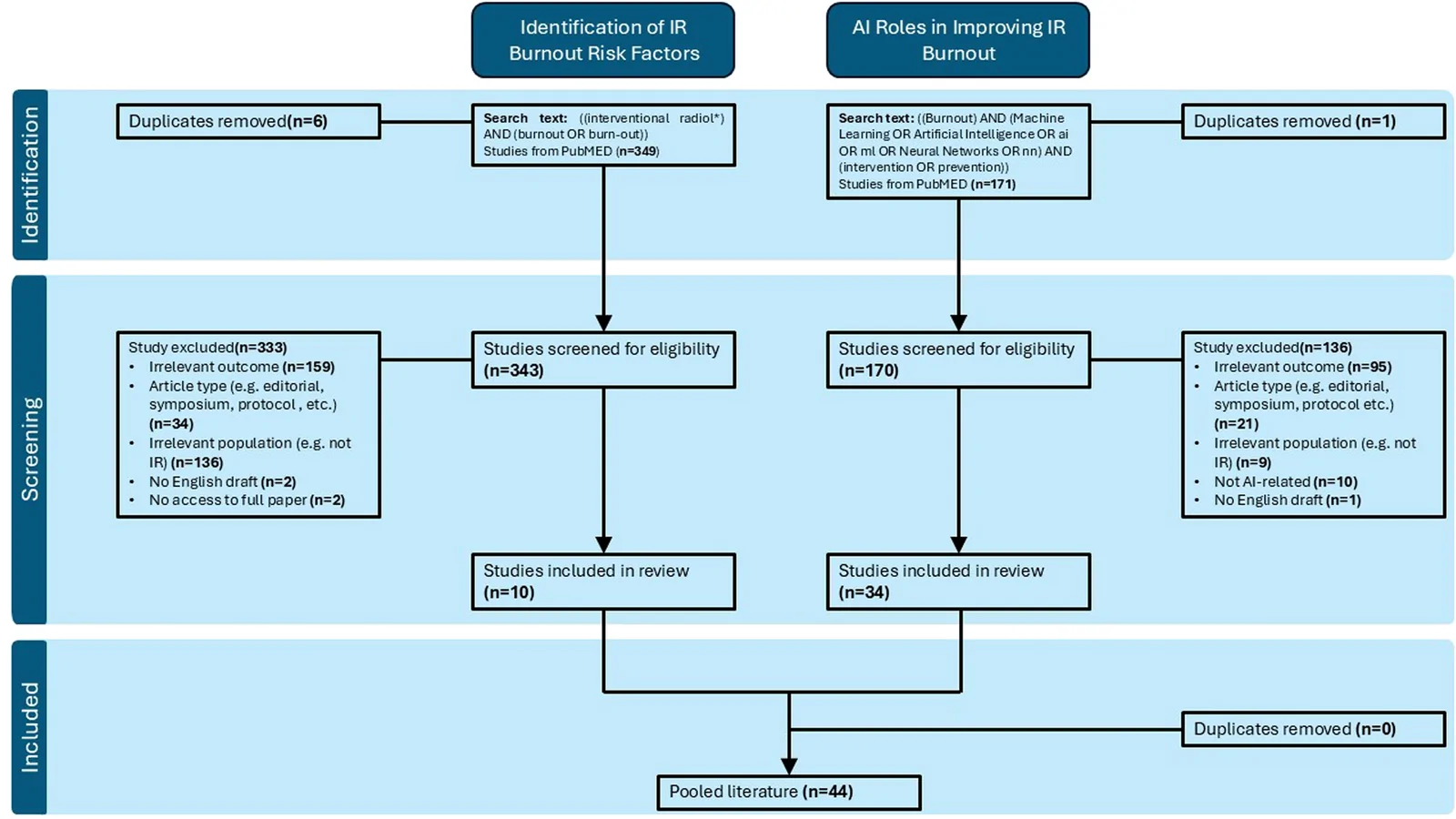
Flow diagram demonstrating the selection criteria of papers being reviewed. The initial search yielded 171 articles on AI and burnout, and 349 articles on burnout in interventional radiology. We included 34 papers on AI and burnout, and 10 papers on burnout in interventional radiology in our literature review. Articles with an irrelevant defined outcome, certain article types (e.g. editorial, symposium), irrelevant population, and no access to English text/full text were excluded

Current literature on burnout challenges in interventional radiology was reviewed. Due to the scarcity of specific AI interventions targeting IR burnout, we ran separate literature searches for AI interventions addressing burnout among physicians. Searches were run on PubMed for articles on the above-mentioned topics from 1 May 1983 to 31 December 2024 using the search text shown in Fig. 2. We included literature reviews and primary studies focusing on the IR population. Duplicates and articles without accessible English full text were excluded. Papers with irrelevant outcomes (e.g. not burnout-related), demographics (e.g. not healthcare-focused), and not AI-related (in the AI intervention search) were also excluded.

### Burden of IR Burnout

Figure 1 summarises the major burnout risk factors in interventional radiology.

### Personal Risk Factors

There is a significant association between female sex and burnout (OR = 2.4) [5]. Female-specific burnout aetiologies and ergonomic burden have been overlooked due to a lack of female participants likely secondary to male predominance in IR [17, 18].

Whether financial incentives would alleviate workload-related burnout is debated. While financial compensation is protective against stress from responding to a call (OR = 0.70) [1], most physicians (72.3%) prefer compensatory time-off over financial compensation [1], highlighting the complex relationship between personal circumstances and burnout risk.

The physical health of interventional radiologists is significantly worse compared to other disciplines [17], with over 60% of interventional radiologists reporting general pain, leading to missed working days (up to 1/3) [18]. Physical pain is correlated to self-reported burnout by inducing physical fatigue [19]. Burnout was associated with undermined sleep quality (*r* = 0.280) [1], leading to consequences such as fatigued driving (39%), road accidents, and medical errors (34%), which also correlated to increased call coverage [20]. There is a higher incidence of anxiety and depression in staff exposed to radiation compared to control groups [21, 22]. Interventional radiologists showed a higher burnout rate than other medical radiation staff, which increases with cumulative radio-exposure years [23]. Prolonged lead coat wearing combined with poor procedure ergonomics results in musculoskeletal disorders in up to 60% IR specialists, leading to increased burnout with a fear for career longevity [24].

There is a significant correlation between burnout and the inability to spend time with family [16], with concerns about work interfering with family life (70%) [1].

Burnout risks increase at early career stages, with challenges balancing clinical responsibilities and training [5, 17], and a lack of sense of accomplishment [1, 17]. Emotional wellness is particularly poor in early careers due to challenges including more demanding work and imposter syndrome, which are alleviated with seniority and career accomplishments [9]. Challenges that affect career growth include unrealistic performance expectations, discipline-based inequalities, and limited growth opportunities [5, 6]. Millennials (i.e. physicians born between 1982 and 2000) are more sensitive to workplace-related stress due to consciousness of rules and high self-demand [5]. Mentorship was suggested as a potential solution to early career burnout [5].

Personality traits such as perfectionism are associated with increased burnout risk in IR [25, 26], whereas traits like resilience and hardiness may be protective [27]. Individuals possessing less effective coping mechanisms or limited social support are more vulnerable to burnout [16, 27, 28].

### Workplace Risk Factors

Workplace-related risk factors relate to resource management within the IR department (Fig. 1), with workload intensity being commonest [29]. Working hours of more than 80 h/week are associated with IR burnout (OR = 7.0) [16].

A significant burnout risk is correlated to high-stake emergency calls. Risk factors include poor organisational workflow, lack of control over call conditions, standard of care paradigm shifts [6], and covering on-call shifts at multiple sites (OR = 1.96) [15]. Weekly hours and out-of-hours cover are identified as significant predictors of emotional exhaustion [9]. Call burden also correlates to a high travel accident risk due to sleep deprivation (OR = 3.36) [20]. A chaotic and hectic work environment was identified as a significant predictor of burnout (OR = 2.09) [30]. These findings underscore the importance of workflow and staffing optimisation in IR departments.

The lack of recognition by other medical specialties is another significant contributor to IR burnout [5]. The feeling of under appreciation by hospital leadership (OR = 4.1, *p* < 0.001) and working with unpleasant colleagues (OR = 1.3) contributed to a 50% prevalence of attrition intention [31]. The perception of being viewed as technicians rather than medical experts can lead to decreased job satisfaction and increased burnout risk [6, 17, 32], highlighting the need for better appreciation of the IR role within the broader medical community.

### System-Level Risk Factors

Evidence suggests a vicious cycle between burnout and malpractice, leading to medicolegal ramifications. IR-related medicolegal lawsuits account for up to a third of all radiology medical lawsuits [33]. Up to 34% of neurointerventionalists reported burnout-related medical errors [20]. High IR malpractice rates highlight the lack of standard IR support and quality assurance in IR [34]. Involvement in medical errors was associated with reduced sleep quality, job confidence, and satisfaction [32]. Peer support sessions are suggested to facilitate disclosure and apology [32].

External factors, such as the COVID-19 pandemic, were also associated with burnout risk (OR = 6.02) [30], highlighting the need for support during times of crisis.

Commonly mentioned management-related challenges include lack of autonomy, administrative pressure, multidisciplinary communication challenges, electronic health records and documentation systems, poor organisational workflow, and lack of control over call conditions [1, 6].

Suggested improvements by interventional radiologists include increasing staff recruitment and improving staff retention by offering better prospects, compensatory leave after on-call responsibilities, balance between diagnostic and interventional workload, abundant time, and resources [5, 9].

### AI for Burnout Prediction and Identification

Artificial intelligence has shown promising performance in identifying and predicting burnout (sensitivity = 84%, AUC = 84%, accuracy = 77%). Feature ranking analysis revealed key predictors of burnout such as mental well-being, higher joint family type, higher age, marital status, weekly duty hours, and HRV parameters [30]. A high-risk signature with varying weights may culminate over a career as the correlation between risk factors and burnout is not necessarily linear [35].

Machine learning models trained on workplace-based data such as EHR-based logs could predict physician MBI scores with demographic correction (AUROC = 0.829) [36]. Algorithms show limited performance when trained solely on EHR measures, suggesting the need to incorporate workplace-specific predictive factors relevant to IR [36]. Nevertheless, AI approaches complement the gold standard MBI in burnout prediction by saving repetitive questionnaire completion.

Another advantage of AI-identified burnout is its time relevance and multimodality. A personalised model predicting empathy and well-being in physicians could identify key contributors such as depressed mood, anxiety, and social connection [35]. Real-time psychological and physiological stress of physicians could be predicted using self-reports and HRV correlated to EHR-based workload assessment [37]. Despite a high individual stress variability (*R*^2^ = 98%), work hour patterns and specific EHR use parameters show strong predictability of physiological stress [37]. Prediction of daily burnout risk (AUROC = 0.6479) could allow early intervention, offering flexible cost-effective fine-tuning with variable sensitivity and specificity specific to workplace needs [38]. A deep learning (DL) model predicting physician departure using EHR data could allow proactive managerial intervention to minimise staff turnover [39].

Another model was trained to analyse clinical notes for physician fatigue (AUC-ROC = 60.1) and unmet healthcare outcomes secondary to burnout. Physicians’ judgement is impaired by 19% between each standard deviation of model-predicted fatigue. A circadian variation was also identified with night-shift staff more exposed to fatigue and emotion distortion by large language model (LLM)-generated notes [40]. Together, AI enables worktime burnout analysis specific to the hour, with a better healthcare outcome.

There is potential for bias in AI models, especially if training data is not diverse or representative of the IR population [41]. Though burnout has objective physiological correlates that can be used in its prediction [30], overreliance on objective factors could risk neglecting subjective experiences (personality, work culture), especially when they may contribute substantially to physician burnout [36]. Extreme response bias can skew the training data of AI models, reducing their practical generalisability [42].

### AI for Alleviating Administrative Burden

**Fig. 3:**
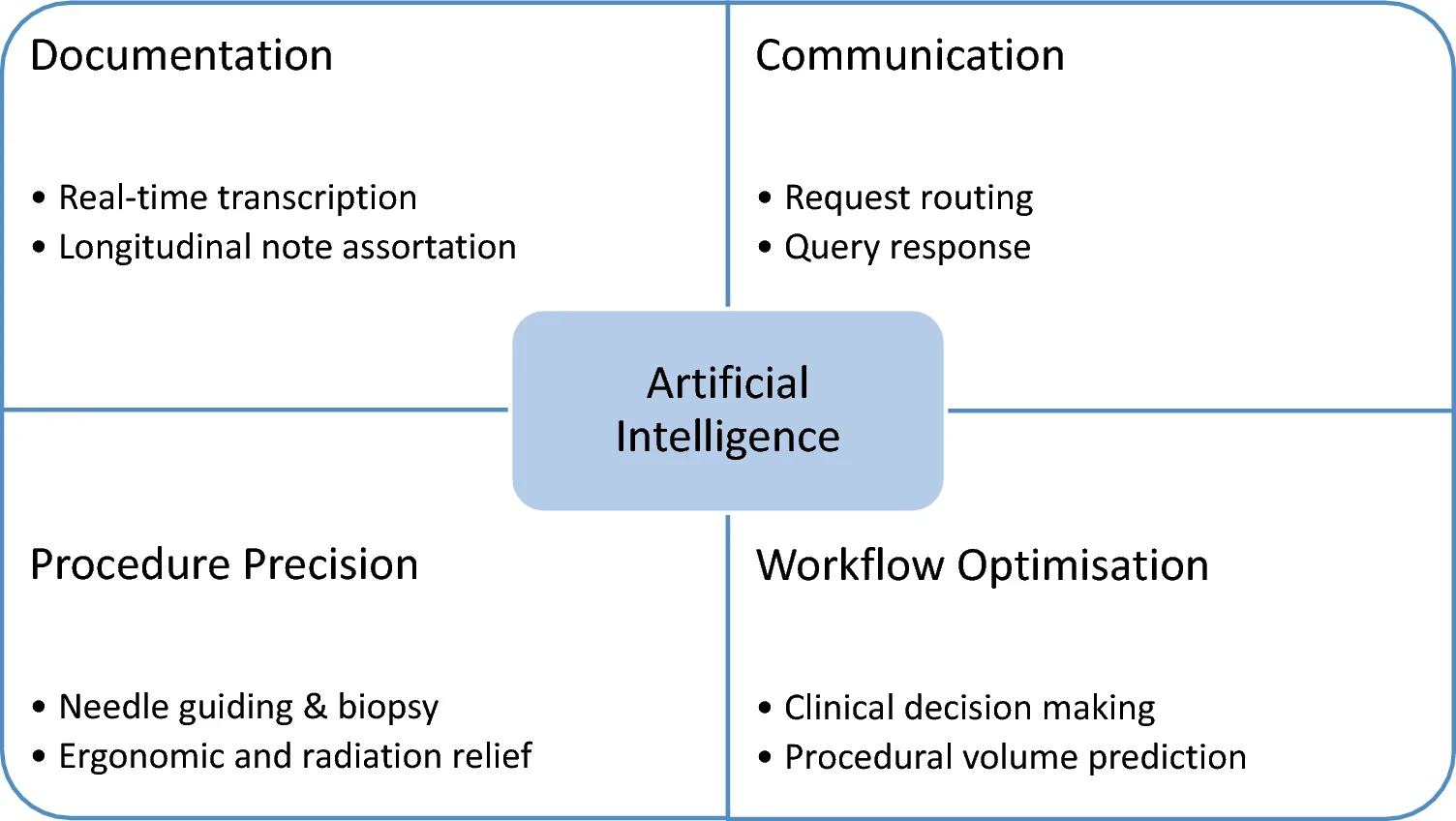
Summary of artificial intelligence-aided identification and relief of radiology burnout. Artificial intelligence has potential in the expedition of clinical documentation with real-time transcription and personalisation with longitudinal note assortation. It also shares a role in communication through inter-disciplinary request routing and automated query response, saving the time and cognitive load of interventional radiologists. Procedures can be made more precise with AI-assisted needle guiding, enabling reduced radiation exposure and ergonomic strain. Artificial intelligence could also optimise workflow by precision protocolling and by enhancing resource allocation according to predicted patient volume

Particular attention has been given to synergising LLMs with electronic health records (EHRs) to address the administrative aspects of medicine, such as documenting patient histories and replying to patient queries (Fig. 3) [11, 43].

### AI for Documentation

‘Patient-centred digital scribes’ incorporating summarising information and signposting to patients could save time by 2–3 times compared to typing and dictation [44].

While showing great promise, the reliability and quality of AI-generated notes remain a concern. A study on the reliability of ChatGPT-4-generated medical notes showed high numbers of errors (23.6 per case) and a modest note quality (Overall PDQI score = 29.7) [45]. Medical hallucinations were reported, with factually incorrect training input leading to misleading documentation [45]. Frameworks such as CREOLA have been developed to guide LLM integration whilst ensuring clinical safety [46].

The multitude of formats used for taking notes strains clinicians sifting through previous notes. Clinicians instead preferred looking at the assessment and plan sections upon review [47]. Notes can also be bloated with irrelevant information [48] with a redundancy of up to 76% [49]. If granted access to longitudinal patient information, LLMs could resolve both problems by assorting patient notes based on relevance, reducing time and cognitive load [50].

### AI for Communication

Commercially available LLMs (ChatGPT-3.5 Turbo and GPT-4) were used to generate draft responses to patient queries to reduce workload and burnout [51]. No changes in reply time were seen, though a significant reduction of 4-item physician task load score (61.31 vs 47.26), and self-perceived clinician time-save were observed [51]. These show that while the objective metric of time may be important for assessing a model’s utility, subjective factors, such as cognitive load, are worth measuring. The overall adoption rate by clinicians was 20% in 5 weeks. The wide heterogeneity of draft adoption rate suggests space for model personalisation to the specific clinical role [51].

Qualitative evaluation of the free-text replies showed common negative remarks including a ‘robotic’ tone, content relevance, effects on workflow and wordiness [51]. Other studies have echoed similar sentiments. For example, despite ChatGPT’s potential in addressing negative enquiries with empathy, information, and recommendations, the model occasionally acted beyond its capacity and lacked reference to longitudinal EHR, resulting in limited personalisation [52].

Responses to patient queries by ChatGPT were mostly comprehensive (71.4%) and factually accurate (83.8%) [53]. Interestingly, physicians’ perceptions of the responses’ empathetic tone did not necessarily reflect patient opinions. In contrast, patients preferred AI-generated responses for their empathy [54, 55]. Future studies are vital to survey patients’ views for more concrete conclusions.

IR-specific examples include a proof-of-concept study of using LLM models for request routing (accuracy = 96.4%), providing an economical solution (0.03USD per request) for communication expedition in a high-stress environment [56].

Healthcare systems will have different requirements based on their patients, limiting the transferability. A less-explored way of fine-tuning a model’s performance is through human feedback and reinforcement learning. This could allow physicians and patients to adjust the outputs generated by the AI. With transferability, models’ knowledge accuracy could improve by as much as 12% over a month [57].

### AI for Workflow and System Optimisation

AI may feasibly emulate the organisational role of medical assistants. A team-based staffing model reduced burnout by 40% within six months [58]. By integrating clinical decision support (CDS) models with EHR systems, AI could help IR specialists make imaging decisions [59]. However, current models as such struggle with analysing complex clinical scenarios. Adaptive decision-making needs to be trained on extensive data input [59]. LLMs are useful in protocolling where decisions for patients with limited information can be optimised with EHR-derived in-depth information [59].

AI models enable risk stratification by predicting transcatheter arterial chemoembolization outcomes based on pre-procedure CT, leading to more specific patient protocolling and improved survival estimation [60–64]. With faster and more accurate subtraction angiography and catheter identification, DL models could potentiate vasculature mapping without contrast [65].

Predictive models for patient volume demonstrating accurate prediction of emergency department volume [66] show IR applicability (Fig. 3), which could optimise staffing and balance elective and emergent case scheduling. A model for image-, speech-, and audio-guided operation assistance trained on IR cases revealed potential for perioperative parameter optimisation, operation duration, and workflow analysis [67]. Similarly, the Pelphix system was trained for phase recognition of fluoroscopy-guided procedures (accuracy = 84%), contributing to workflow prediction and identification of standard workflow deviation, although IR-specific data is lacking [68]. Machine-learning-based surgical workload prediction led to reductions in error, presurgical resource consumption, and staff wait time [69].

### AI for Procedure Precision

AI models provide precise biopsy-taking, alleviating stress during such invasive procedures. Robotic-assisted CT-guided lung biopsies showed similar deviation compared to interventional radiologists with 5 years of experience, but had shorter procedure time [70], reducing radiation exposure and ergonomic stress. A fully-automated MRI-guided prostate biopsy achieved needle localisation at human expert performance [71]. There is a lack of training data in ultrasound-based models [72]. However, synthetic data generated from real-life clinical scenarios could train models for needle localisation in ultrasound procedures, saving time from extensive manual annotations [72].

AI models could potentially tackle the physical and psychological stress of radiation exposure. AI-enabled navigation systems could achieve adequate accuracy in procedures (microwave ablation, lung biopsies) with significantly lower radiation exposure to staff [73–75]. AI and robotic techniques could also relieve ergonomic stress among IR specialists. The use of a flexible C-arm system significantly reduced table repositioning, tube dislodgement, and operator discomfort, without compromising patient safety [76]. Future studies could explore the direct improvement in physicians’ well-being including reduced burnout scores or fewer sick leaves due to ergonomic pain or radiation exposure.

## Discussion and Future Directions

Analysis of burnout risk factors included findings from broader physician or surgical cohorts due to the scarcity of IR-specific burnout studies. The assumption that burnout risk factors could be extrapolated across medical specialties may limit the direct applicability of the findings to IR.

**Fig. 4:**
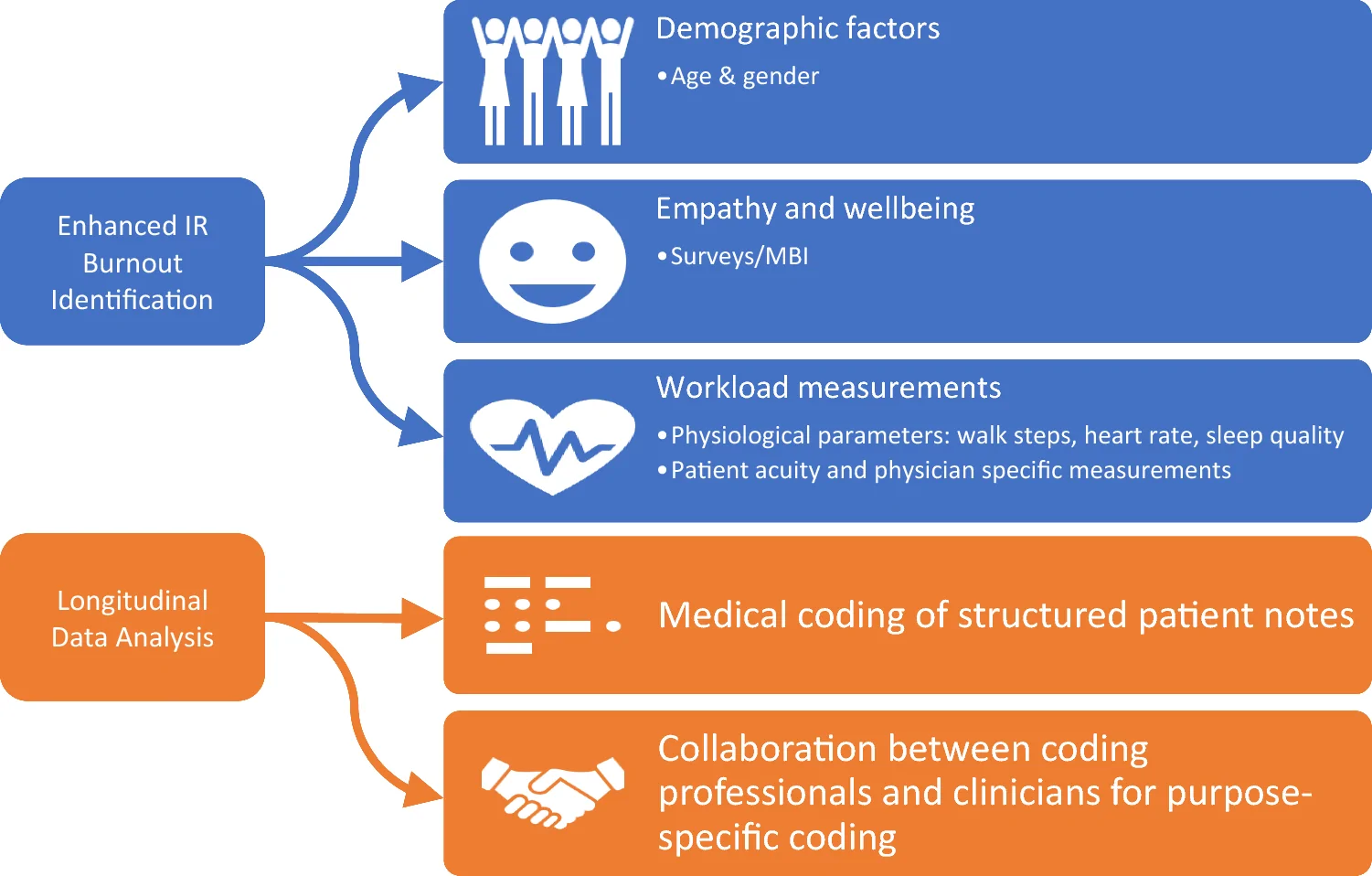
Future Directions of AI as a Solution to Burnout in Interventional Radiology. The identification and relief of burnout in interventional radiology could be personalised with multimodal data input, including demographic factors, empathy and well-being assessments (e.g. MBI-derived parameters), and workload measurements including physiological parameters, and workplace-specific measurements. Longitudinal access to healthcare data for better workload relief involves improved medical coding of electronic healthcare records and close collaboration between coding professionals and clinicians

Future efforts to improve IR burnout identification are warranted (Fig. 4).

Main challenges behind using the 22-item MBI-HS as the gold standard are question redundancy and lack of physiological association. Some single items in the MBI are almost as predictive of burnout as the full survey, suggesting the possibility for a more concise and thus accessible routine self-assessment [77, 78]. Similarly, the MBI-HS has shown an equivalent predictive value as the 7-item Oldenburg Burnout Inventory [78]. Demographic factors such as age and gender are strong indicators of burnout, and real-time assessment of empathy and well-being allows more rigorous evaluation of individuals’ psychological needs. Workload assessments specific to IR could also be explored. The BROWNIE study attempts to combine physiological parameters (walk steps, heart rate, and sleep quality), psychological surveys, and workplace factors in a dynamic Bayesian network to predict burnout in nursing staff [13]. Given the different responsibilities between physicians and nurses, a physician-specific measurement of physician workload could be explored, which may include detailed measurements of workload, ergonomic challenges, and emergency calls. A platform incorporating the above factors would help identify burnout and facilitate burnout support.

Most AI solutions discussed above exist at a low technology readiness level and remain to be evaluated in real-life environments. It is challenging to foresee burnout improvement following AI implementation due to the indirect link between workload alleviation and burnout prevalence. AI tools’ dependence on computing power may limit their technical and financial accessibility. Furthermore, AI tools focusing on objective factors have limited applicability due to human factors (personality traits, work environment) which also play a significant role in the multifactorial burnout challenge [36]. Intervention to objectively predicted burnout could be limited by the low participation rate witnessed among radiologists [79]. Longitudinal studies incorporating cost-effectiveness analyses are warranted to determine whether workload optimisation could translate into sustainable well-being improvements.

Addressing workload challenges to alleviate burnout incidence requires managerial and technical advancements (Fig. 4). The lack of AI insight into longitudinal data limits the models’ ability to personalise responses. So far, AI models are able to analyse longitudinal medical codes such as the International Classification of Disease [80]. Attempts were made to standardise unstructured patient notes for AI analysis [80]. Combining structured note processing with data analysis could improve consultation efficiency and expedite necessary interventions. However, challenges of automating priority coding algorithms, often based on clinical expertise and clinical purposes, as well as fine-tuning algorithms for clinical or research emphases, are both prominent. On a systemic level, greater collaboration between coding professionals and clinicians is required [80].

## Conclusion

IR faces significant burnout challenges due to personal, workplace, and systemic factors. AI emerges as a promising tool offering solutions to burnout detection, workflow optimisation, and administrative burden relief. However, AI implementation into practice must be approached cautiously, considering the need for speciality-specific adaptations. Future research should focus on developing IR-specific AI models and rigorously evaluating their real-world impact.
